# Training undergraduate research assistants with an outcome-oriented and skill-based mentoring strategy

**DOI:** 10.1107/S2059798322005861

**Published:** 2022-07-14

**Authors:** Dennis Della Corte, Connor J. Morris, Wendy M. Billings, Jacob Stern, Austin J. Jarrett, Bryce Hedelius, Adam Bennion

**Affiliations:** a Department of Physics and Astronomy, Brigham Young University, Provo, Utah, USA

**Keywords:** mentoring, undergraduates, CASP, protein structure prediction, deep learning

## Abstract

Effective mentoring of undergraduate students is a growing requirement for the promotion of faculty at many universities. It is often challenging for young investigators to define a successful mentoring strategy, partially due to the absence of a broadly accepted definition of what mentoring should entail. To overcome this, an outcome-oriented mentoring framework was developed and used with more than 25 students over three years. It was found that a systematic mentoring approach can help students quickly realize their scientific potential and result in meaningful contributions to science. This report especially shows how the Critical Assessment of Protein Structure Prediction (CASP14) challenge was used to amplify student research efforts. As a result of this challenge, multiple publications, presentations and scholarships were awarded to the participating students. The mentoring framework continues to see much success in allowing undergraduate students, including students from underrepresented groups, to foster scientific talent and make meaningful contributions to the scientific community.

## Abstract 2

It is found that research mentoring of undergraduate students can result in meaningful contributions to scientific disciplines. Here, an outcome-oriented, skill-based mentoring strategy is proposed and evaluated in the context of the CASP14 community challenge.

## 1. Introduction

An increasing number of universities expect faculty to enrich the undergraduate curriculum by providing meaningful research opportunities for enrolled students. Recent reports indicate that mentoring relationships have many direct benefits (Gee & Popper, 2017 ▸; Gee, 2019 ▸). Marshall and coworkers state that mentored students acquired benefits in relational knowledge, self-awareness and career development (Marshall *et al.*, 2021 ▸). Goodman-Wilson claims that mentoring relationships can be indicative of students’ psychological challenges, such as depression or social anxiety, therefore providing opportunities to identify struggling students (Goodman-Wilson, 2021 ▸). Morales and coworkers found that mentoring can also be an effective tool to support diverse students (Morales *et al.*, 2021 ▸).

It is surprising that despite the large amount of evidence for positive mentoring results, little can be found in the published literature on effective research mentoring of undergraduate students. Further, reviews found as many as 50 different definitions and limited progress in building a consensus in definition, theory and methods for mentoring in general (Gershenfeld, 2014 ▸). In the absence of a common definition of mentoring, we follow the suggestion of Koch and coworkers and define a mentoring strategy based on the goals that mentoring is supposed to achieve (Koch & Johnson, 2000 ▸). We hypothesize that a skill-focused mentoring program will enable meaningful research outcomes.

The relationship between student and faculty is frequently called mentoring, with the supervisor taking the role of a trusted advisor or mentor and the student that of a learning protégé or mentee. While the origins of the term ‘mentoring’ go back to the Greek hero Odysseus, who entrusted his son Telemachus to a wise counselor named Mentor, the modern definition of this term is still under debate (Koch & Johnson, 2000 ▸). Some of the common elements found in successful mentoring programs reportedly include matching, preparation, interaction and evaluation (Gannon & Maher, 2012 ▸).

In a thoughtful summary of the progress of student mentoring, Gonzales argues that the boundaries between graduate and undergraduate research are becoming blurred as the US research university evolves (González, 2001 ▸). This is driven by the needs of a knowledge-based global economy, where students with research experience show an increased ability to adapt quickly to new situations and to solve difficult problems.

Adding research requirements to undergraduate experiences carries an inherent risk of distracting students from core class objectives. However, Heager and Fresquez found that students mentored in research had significantly higher cumulative grade point averages and similar graduation rates as a matched set of peers (Haeger & Fresquez, 2016 ▸). Johnson and coworkers discussed the significant costs associated with an effective mentoring program for faculty in terms of time devoted to mentoring, expenditure of emotional energy and relationship-induced stress (Johnson *et al.*, 2015 ▸). Johnson and coworkers further warn that time spent mentoring may result in increased risk of faculty burnout, decreased productivity and difficulty with promotion milestones.

It is apparent that the current literature applauds the efforts of faculty members who elect to be mentors and identifies many benefits for the mentored students; however, the reported risks for these faculty members could carry severe implications. Given the pressures of an early scientific career and a ticking tenure clock, the increasing mentoring expectations for young faculty members can appear overwhelmingly stressful. Nevertheless, if the outcomes of a mentoring strategy are aligned with the goals of mentor and mentee, success and deep satisfaction can result. We are of the opinion that the mentoring relationship between faculty members at research universities and undergraduates involved in their research programs is a key differentiator between failed and successful mentoring. To support this, the objective of the mentoring strategy presented here is to develop students into collab­orators and not only to advise them on course material or study/career plans.

The current literature provides little evidence that research-based mentoring offers long-term benefits to either mentors or mentees. While general classroom and social mentoring is frequently a subject of discussion, little is known about effective ways to use mentoring for the integration of undergraduates into research programs. This report sets out to provide a unique perspective on the relationship between a faculty member and a team of undergraduates. We provide a case study that highlights an outcome-oriented skill-based mentoring (OOSBM) strategy that can be adapted at other institutes and evaluate the efficacy of this strategy through a retrospective analysis covering two years. We claim that an OOSBM strategy can help undergraduate students to rapidly assess their research potential and to contribute meaningfully to their scientific discipline. This in turn increases the productivity of the mentor’s laboratory and thereby supports the achievement of other academic goals.

## 2. Framework

### 2.1. Outcome-oriented skill-based undergraduate mentoring strategy

**Figure 1:**
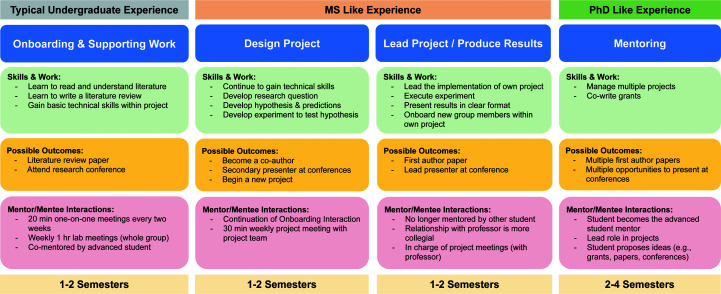
Outcome-oriented skill-based mentoring strategy for undergraduate research experiences.

We propose a novel outcome-oriented and skill-based mentoring (OOSBM) strategy as visually summarized in Fig. 1 ▸. Aspects of this strategy first emerged naturally in the Della Corte Laboratory at Brigham Young University (BYU) and were later formalized into this reusable framework. Each phase serves as a meaningful training round but also as a filter to rapidly identify students with research aptitude. The aim is to enable undergraduates to obtain rich research experience comparable to that of graduate programs to best prepare them for industrial and academic careers after graduation. It is aligned with the academic age of a student in a typical four-year bachelors’ program in the US: freshman, sophomore, junior and senior. Students may start with the onboarding phase at any point in their program, but the earlier that they enter the further they can proceed. For this model to work, students are expected to work weekly between ten and 20 hours in the Winter and Fall semesters and between 20 and 40 hours in the Spring and Summer semesters. These hours typically do not contribute to the credit hours taken towards a program but are extracurricular. However, some programs allow students to take some research credit as an elective and this option is encouraged where available.

Study plans in various STEM departments across the US suggest that most programs limit the exposure of undergraduates to research to the last one or two semesters of their degree, sometimes in conjunction with a senior thesis (Trosset & Weisler, 2018 ▸). This is unfortunate, as students are barely able to comprehend scientific literature and perform simple tasks in research projects after completion of this phase. While publications with undergraduate co-authors are sometimes the results of such limited research mentoring, first-author undergraduate papers are nearly impossible. The OOSBM strategy proposes that starting students out earlier could allow them the necessary time to build on their onboarding knowledge and design their own projects.

The onboarding phase has the goal of letting students contribute to a scientific publication. It is important for students to see early on where research should lead and teaming them up with a more senior student in the laboratory is often beneficial. The typical task for students in this stage is a systematic literature review, either for a project proposal, review paper or introduction/discussion section. The OOSBM strategy builds on this by asking undergraduates to conduct a more substantial literature search (for example reading between 100 and 150 research papers). We believe that this will differentiate research-oriented students from laboratory tourists (*i.e.* students who will make a significant contribution compared with those who are more interested in seeing the sights of your laboratory group), who typically drop out of the group within the first semester. Ideally, students are awarded a paid research position at this stage to offset social differences. Other activities at this stage could involve taking a scientific writing course, learning how to prepare scientific figures and running basic analysis on data sets provided by collaborators or group members. A student who has successfully contributed to a publication and has developed the necessary literature comprehension is then tasked to write their own research proposal.

Within the design phase of a project, students use the literature review that they developed during the onboarding phase to formulate a hypothesis for original follow-up work. Building on the methods that they are now acquainted with, they can choose the experimental setup most suitable to test their hypothesis and initiate first proofs of principle. In this phase, their responsibilities are comparable to that of a masters student in a typical graduate program, as they prepare and defend a prospectus at a weekly group meeting, create a project plan and prepare for leading the implementation efforts.

Within the project lead phase, students could team up with other undergraduates for small aspects of the work (such as running specific simulations or training machine-learning networks), but overall they would assume the role of a project lead and secure a first authorship. We expect that most students would not be likely to make it through this stage before graduation, but if successful they could come out of their undergraduate studies with two to three publications and substantial independence in conducting research.

Those students that started early enough and showed exceptional talent (the top 5%) can progress to the fourth level of a mentor. This is a PhD-like experience where they start to train and lead a set of other undergraduates (who are typically at levels 1 and 2) on a variety of projects. They would also support the PI in the process of writing grants and may manage to gain co-authorships on multiple papers.

### 2.2. Elements of research projects conducive to the success of novel mentoring strategy

While working with over 25 undergraduate research assistants over the course of three years, we found that some projects are more conducive than others in reaching the objectives of an OOSMB strategy. In our program, successful undergraduates have been able to graduate with over five publications and secured high-profile scholarships and grants for graduate school (for examples of review papers, see Section 4.2). While each scientific discipline has their own challenges and benefits, we suggest that the following criteria be considered as a way to evaluate new projects for suitability as undergraduate-driven research projects.

#### 2.2.1. Accessible literature

Students should be able to easily identify the most relevant articles for their project. Having access to recent review articles, specific journals or special editions devoted to the question that they are setting out to research provides invaluable insights. Students learn not only subject-matter expertise, but can also learn patterns of successful publications in their respective field. Good authors are great readers; therefore, providing students with the best literature in the field is critical for success. In addition, frequent conversations in the team (either during group meetings or via electronic communication tools such as Slack, Discord or Microsoft Teams) are essential to help new laboratory members to identify relevant literature.

#### 2.2.2. Clear problem definitions

Unexperienced researchers often struggle with the formulation of a testable hypothesis. Some established research fields feature existent well defined problems. Tackling a problem with a history provides students with meaningful context and often allows them to grasp the importance of their work more clearly than an offshoot explorational project would.

#### 2.2.3. Crisp timelines

Students battle with conflicting priorities, especially during testing-heavy semesters and due to increasingly complex personal lives (for example, many undergraduates at BYU marry young and frequently have children before graduation). Selecting projects with externally forced timelines can counteract the tendency to procrastinate. While this can also put more stress on students, it is a valuable lesson for future projects, when external stakeholders will require results independent of personal challenges.

#### 2.2.4. Plannable

Occasionally, *ad hoc* projects can be highly rewarding, but for undergraduates a project plan is most essential. If students do not know what is expected by when, they can easily get lost or sidetracked in details or distractions. Creating an aligned Gantt chart and having frequent meetings are important. However, if external factors mandate a plan it tends to be easier to motivate students than internal deadlines would.

#### 2.2.5. An active research community for networking

Publishing a paper can be very rewarding, but discussing it with other professionals leaves the greatest impact on a student’s self-perception. Access to focused conferences or interaction with a network of collaborators/partners is an essential criterion for students seeking to find their role in the scientific community.

#### 2.2.6. Potential for high impact

A project without future impact has limited meaning to a student. While certain foundational questions are often of great interest to a seasoned researcher, the minute details are often illusive to the untrained student. Especially for motivation in a large research group, it is important for a student to be able to explain the importance of their respective project in clear and brisk terms. The future employability of students also benefits when they can deliver elevator pitches about their work.

## 3. Case-study description

### 3.1. Host institute

Brigham Young University is an R2 research university (*i.e.* a university with high research activity) focusing on undergraduate education. The Della Corte Laboratory is part of the Physics and Astronomy department and hosts the interdisciplinary Consortium of Molecular Design. Within this consortium, students from Physics, Chemistry, Biology, Computer Science and Engineering collaborate on diverse projects involving protein engineering, drug discovery and laboratory informatics. The Della Corte Laboratory was started in October 2018 and currently hosts two affiliate faculty members, three graduate students and 15 undergraduate students. In total, over 30 undergraduates have been mentored within this laboratory. This report focuses on the four students mentored in context of the CASP14 challenge.

### 3.2. Participants

For this report, a team of four undergraduate students was mentored for over three years according to the OOSBM method in the context of a community challenge. Table 1 ▸ provides details of the students involved. This report focuses on the specific mentoring opportunity offered by community challenges. While all students in the group are mentored according to the described framework, this report focuses on those that participated in CASP14. The research team from whom data were collected are also authors of this article and no written consent for their inclusion was required.

### 3.3. Description of community challenges

We believe that community challenges per definition check off most of the criteria outlined previously. These communities are typically organized around well established research questions, have their own conferences or proceeding reports and host experiments that often follow plannable patterns. We provide an overview of some of the community challenges in computational biology in Table 2 ▸ (Boutros *et al.*, 2014 ▸). Similar lists can be found for various other disciplines.

We selected the CASP challenge as a testing ground for our OOSBM strategy. The CASP challenge is a highly visible community event and has been held at regular intervals every two years since 1994, with the most recent iteration, CASP14, finishing in December 2020 (Pereira *et al.*, 2021 ▸). The aim of this challenge is to benchmark methods that can be used to predict the folded structure of proteins and related objectives from limited information, such as amino-acid sequences. For this, a set of experimentally solved (but not yet published) protein structures are shared by laboratories with the assessment center. CASP then releases, over a period of multiple months, information about these structures, called prediction targets. A large community, often over 100 registered groups, has a limited time window to make predictions, after which they share the results back for assessment. In a concluding conference, typically in December of the same year, the results of the different experiments are shared, and selected groups present their methods. The following year, a special issue in the journal *Proteins* is dedicated to reports from the best-performing groups and general experimental assessments.

### 3.4. Data sources and analysis

To better understand the structure and perceived outcomes of our mentoring program, we collected data in the form of participant and group laboratory journals, project calendars and semi-structured interviews of the laboratory group members (see Table 3 ▸; Weiss, 1995 ▸). We used the project calendars and laboratory journals to catalog how often the group met, the purpose of each meeting (for example data analysis, progress updates, social) and to obtain a sense of the productivity and timeliness of the group’s progress. We also used semi-structured interviews to understand the perspectives of the participants. Dr Bennion (a science education researcher in the Department of Physics at BYU) wrote the protocol (see supporting information) and conducted the interviews. The interviews were conducted in person when possible and over Zoom when necessary. We recorded the interviews and transcribed them for coding purposes. We interviewed each participant of the laboratory group that participated in CASP. Because this is a self-study (all of the participants are authors of the paper) no formal consent was required.

In our analysis, we organized the data from the interviews into several categories (for example Skills and Outcomes, Why Do Research, Contributions *etc.*). Within these general categories, we developed themes through open coding (Maxwell, 2012 ▸), a process that involved finding patterns based on the comments of the respondents and refining these themes into codes by the trends found across the data (see Tables 4 ▸ and 5 ▸ for descriptions and examples of each theme). To test the legitimacy of the coding, we presented the themes to the participants to check our interpretation of the interviews. We used the laboratory journals and project calendars to construct timelines for the project. Data from these analyses are presented in the findings below.

## 4. Case-study results

### 4.1. Results from applying the mentoring strategy to CASP for the participants

Analysis of the interviews conducted with the project participants allowed the identification of specific skills that the participants claimed to have developed. The four students are co-authors on one, three, four and five articles published while students at BYU, and all presented posters or gave talks at international conferences. We list recurring themes with concrete examples from the interview in Table 4 ▸. Besides the necessary technical and literacy skills, we observed that students pointed out the development of communication, networking and project-planning skills. As the challenge posed external deadlines the resulting stress levels were occasionally difficult for the students to manage. This became apparent during daily early morning meetings, where some students would either not show up or be rather agitated. We also found that the CASP conference enabled the presentations and interactions of the students with the domain experts that was hoped for. We find that students report the development of various skills that agree with the objectives defined in the OOSBM strategy.

### 4.2. Preparing for, executing and evaluating the CASP14 challenge

**Figure 2:**
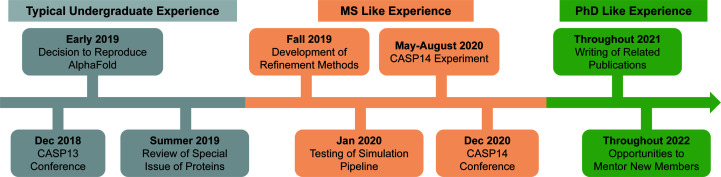
Mapping the CASP timeline to our mentoring strategy.

The timeline of the CASP14 challenge is displayed in Fig. 2 ▸ and mapped against the proposed OOSBM strategy. In late 2018, our team began the onboarding phase by attending the CASP13 conference and collecting relevant literature. After about six months, we understood the best methods in the field and designed new approaches based on them (Billings *et al.*, 2019 ▸, 2021 ▸; Stern *et al.*, 2021 ▸). Before the official CASP14 challenge began, the assessment center launched a community-wide experiment to help with the structure determination of COVID-19-related proteins (Kryshtafovych *et al.*, 2021 ▸). This presented itself as an excellent opportunity to test the data pipeline and submission scripts for our group.

The real CASP14 challenge began in May 2020 with the release of the first protein sequence. We made predictions for all targets in the Free Modeling, Refinement, Contact, Distance and Experimentally Assisted test categories (for detailed definitions, see Kinch *et al.*, 2021 ▸). The three undergraduates who were responsible for running the predictions worked contracts of between 20 and 40 h per week. Each morning, we met at 8 a.m. through Zoom to go over all the various targets and tracked progress in a multi-stage pipeline on a Google sheet. Sometimes these discussions would take only a few minutes, but at times multiple challenging targets would require us to spend hours going over the details.

A remote conference in December 2020 marked the end of the CASP14 experiment. At this conference, each participant from the Della Corte Laboratory presented posters on the methods that they had used during the experiment and others developed afterwards (Morris *et al.*, 2020*a* ▸,*b* ▸, 2021 ▸). We were also invited to contribute to the Refinement Roundtable discussion and students held related presentations at a variety of other conferences (Morris, 2021 ▸). The networking opportunities with leaders in the field from both academia and industry proved very valuable to the students and helped them to realize their scientific potential.

**Figure 3:**
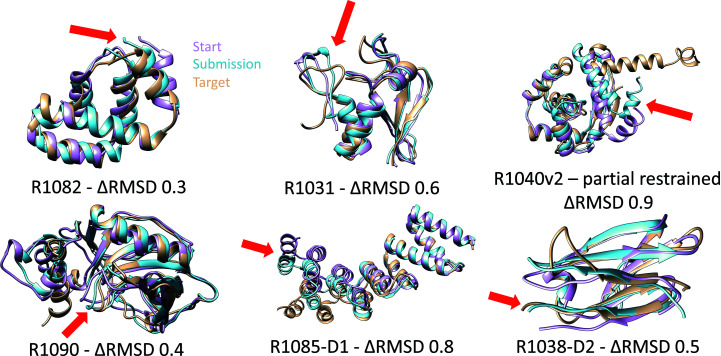
Visualization of successful refinement targets by the Della Corte Laboratory at CASP14. The goal of refinement is to move the pink start structure closer to the golden target structure. Blue is an improved submission, where improvement is expressed in delta root-mean-square-deviation (ΔRMSD) between the target and start versus target and submission C^α^-atom positions of the protein structures.

Within the Refinement category, we were one of only three laboratories that on average improved the predicted protein structures. The goal of the refinement section in CASP is to improve a good protein structure prediction further. These improvements are often very small, as shown for a set of targets in Fig. 3 ▸. A detailed description of our refinement protocol and its strengths and weaknesses will be reported in a separate manuscript.

### 4.3. Evaluating the suitability of the CASP community challenge as an undergraduate research project

The undergraduates appreciated leveraging community challenges for several reasons. Table 5 ▸ outlines the benefits that they discussed in their interviews as themes or conditions that can generally be applied by other research groups who are seeking to engage their undergraduates in a similar way. We note that the specific features, as mentioned by the students, align very well with the above-defined criteria for ideal undergraduate projects. In particular, the commentary on how the challenge helped the students to understand their scientific potential and to plan out their careers is deemed to be a great success of the OOSBM strategy.

## 5. Discussion and conclusions

Given the large differences between existing mentoring definitions (Gershenfeld, 2014 ▸) and the absence of a well established scheme for mentoring undergraduate research assistants, we proposed a novel OOSBM strategy and tested it over the course of three years in the context of the CASP community challenge. The mentoring relationship between the students and faculty has shown multiple direct benefits, as suggested elsewhere (Gee & Popper, 2017 ▸; Gee, 2019 ▸). We particularly agree with Marshall and coworkers, and find that the students achieved a greater degree of self-awareness and career development as they understood their potential as researchers and their specific research areas better (Marshall *et al.*, 2021 ▸; Mello *et al.*, 2017 ▸). In this project and in other mentoring activities since, we have found evidence for the claims of Morales and coworkers that research mentoring can overcome challenges for diverse student groups (for example gender, culture, ethnicity) by giving them a common objective that unifies (Morales *et al.*, 2021 ▸). The successfully mentored research students all hold very high grade point averages and have been able to maintain graduation timelines, as also reported by Haeger & Fresquez (2016 ▸).

Contrary to the emotional, time and productivity cost reported by Johnson and coworkers, our OOSBM strategy has proven highly rewarding for the faculty member involved; it fostered deep friendships on a personal plane as well as resulting in an increased number of published articles on a professional level (Johnson *et al.*, 2015 ▸). This might be partially due to the conducive environment for undergraduate research at BYU, where student salaries were paid by college funds, or the generally lower tenure requirements compared with some R1 schools (*i.e.* doctoral universities with very high research activity) in terms of the impact factor of published articles and/or the size of secured external grants. However, following González (2001 ▸) and realizing that the boundaries between graduate and undergraduate research are becoming blurred in US research universities, we anticipate that research laboratories at R1 institutes can also access the benefits of the OOSBM strategy. Creating paid undergraduate research opportunities is also a powerful lever to overcome socioeconomic differences that might otherwise force some highly gifted students to pursue employment outside the university.

Undergraduate research mentoring is expected to remain a growing responsibility for instructors at many universities and we found that it requires conscientious planning to be successful. Our proposed strategy can help undergraduates to quickly explore their scientific aptitude. The point-based evidence from applying the strategy to CASP suggests that meaningful undergraduate mentoring can be personally and professionally rewarding to mentors and mentees. We found that community challenges provide a strong framework for meaningful undergraduate research projects.

We suggest that community challenges such as CASP can provide the following benefits.

(i) It is a plannable event that requires the most time during the summer months, which are often not as class-load intensive.
(ii) The recent progress in the field can easily be read up on from a consolidating journal.
(iii) A large set of previous CASP experiment information is available for benchmarking new methods.
(iv) The gamification elements of leaderboards and ‘winners’ creates a highly motivating environment.
(v) The conference allows poster submissions from all participating groups, which gives students the chance to meaningfully contribute to a networking event.
(vi) If successful, invited publications enable students to become co-authors on rapidly published articles.

We caution that some aspects of community challenges also bear an intrinsic risk, as expressed by one of our participants during the interview:

I think if [the competition] is the only thing the science is being developed for, that could be less helpful because if you don’t do well … I don’t know quite how the students would feel about it … if it’s solely developed for the competition.

We agree that while CASP was in general a successful event for our students, other groups with less success might have lost their motivation. Nevertheless, we consider this to be an ideal preparation for industry projects, which are frequently accompanied by high stress levels and subject to unexpected changes or abrupt terminations.

The key is to help students look beyond the competition and to help them realize after the event how much personal growth occurred during it, regardless of the overall outcome obtained by the group. Allowing personal feedback and reflection after an undergraduate project is an essential part of the learning experience, especially for students that are used to measuring their academic value solely by numeric scales such as grade point averages. We found that a small party with a nice dinner at a local restaurant provided a great closure event.

We recommend principal investigators interested in involv­ing undergraduates to use a systematic model, such as the OOSBM strategy introduced here. It provides clear guidance to the mentor and mentee, and is focused on producing outcomes of benefit for students and faculty alike. Further, we encourage institutions to consider using available funds more proactively in support of undergraduate research assistants; they tend to cost a lot less than graduate students but can yield comparable results. Finally, while the OOSBM strategy could be effective outside events such as CASP, we encourage researchers to explore community challenges in their respective fields as they have been identified as ideal testing grounds for novel mentoring models.

In summary, well mentored undergraduate research opportunities, such as CASP, challenge students to grow outside their comfort zone. These opportunities do not need to fit into a specific program of study but are suggested to be extracurricular, with similar benefits to those discussed by Mello *et al.* (2021 ▸). In our case, the methods developed by the students contributed to one category at the top of the field. Associated publications and network access helped the students see their value as researchers and contributors. Especially for underrepresented groups, such as females and minorities, this early exposure to meaningful research can help them realize their potential and lead to a successful scientific career [for example, three female laboratory members have been awarded with the prestigious Goldwater scholarship (Kettler & Puryear, 2021 ▸) in 2020, 2021 and 2022]. Using a systematic mentoring strategy such as the OOSBM strategy can provide a framework to enhance undergraduate education to the next level.

## Supplementary Material

Interview protocol. DOI: 10.1107/S2059798322005861/ai5005sup1.pdf

## Figures and Tables

**Table 1:** Students involved in this study who contributed to CASP14

Name	Joined research group	Gender	Major	Current program
Mary	October 2018	Female	Chemistry	Chemistry PhD at Berkeley
Bill	October 2018	Male	Applied Physics, pre-med	Bachelor of Physics BYU, MD/PhD UCLA
Joe	October 2018	Male	Physics	MS Physics BYU
Kyle	December 2020	Male	Computer Science	PhD Computer Science BYU

**Table 2:** Overview of community challenges in computational biology (adapted from Boutros *et al.*, 2014 ▸)

Challenge	Scope	Assessment type	Organizers	Website
CAFA	Protein function prediction	Objective scoring	Community collaboration	http://biofunctionprediction.org/node/8
CAGI	Systems biology	Objective scoring	UC Berkeley/University of Maryland	http://genomeinterpretation.org/
CAPRI	Protein docking	Objective scoring	Community collaboration	https://www.ebi.ac.uk/pdbe/complex-pred/capri/
CASP	Structure prediction	Objective scoring	Community collaboration	http://predictioncenter.org/
CACHE	Drug discovery	Objective scoring	Community/industry collaboration	https://cache-challenge.org/
ChaLearn	Machine learning	Objective scoring	ChaLearn Organization (not for profit)	http://www.chalearn.org/
DREAM	Network inference and systems biology	Objective scoring	Community collaboration and Sage Bionetworks	https://dreamchallenges.org/
FlowCAP	Flow cytometry analysis	Objective scoring	Community collaboration	http://flowcap.flowsite.org/
IGCG–TCGA DREAM Somatic Mutation Calling	Sequence analysis	Objective evaluation	Community collaboration and Sage Bionetworks	https://www.synapse.org/#!Synapse:syn312572
Kaggle	Topics in various industries	Objective scoring and evaluation by judges	Commercial platform	http://www.kaggle.com/
X-Prize	Technology	Evaluation by judges	X-Prize Organization (not for profit)	http://www.xprize.org/
2021 Ligand Model Challenge	Structure determination	Objective evaluation	Community collaboration	https://challenges.emdataresource.org/?q=2021-model-challenge

**Table 3:** Data sources used for analysis

Data source	Amount	Description
Laboratory journals	3	Digital or handwritten accounts of the work the participants engaged in as they moved through the CASP project as a part of the mentoring program
Project calendars	6	These calendars detail how often the laboratory group met and what the objective of each meeting was for a given semester
Semi-structured interviews	5	Interviews (∼25 min) of the undergraduates and mentor professor who participated in the research mentoring program. The interviews focused on learning about the skills developed and outcomes achieved because of the mentoring strategy.

**Table 4:** Skills reported by participants in post-experiment interviews

Skill	Description	Example
Technical	Students gain technical skills that are discipline-specific as they work in various group projects	*Definitely coding skills. I’ve done … Python coding and batch scripting before I started … but definitely most of my training now has been just from research.* (Bill)
Understanding literature	Students learn how to read, interpret and write literature as they begin investigating their own field of interest. They are guided in this during the regular laboratory group meetings as they present and discuss their findings.	*I think probably the biggest skill is diving into a field where I do not have experience and identifying what I do not know in that field. Like, just opening up a paper, seeing a bunch of terms that I don’t understand, but having a starting point for knowing how to quickly come up to date on research and on a particular niche of research in the field.* (Kyle)
Communication and networking	Students learn how to reach out to experts in the field and to discuss issues that are meaningful to their study. This often happens and is developed at conferences.	*The ability to go find resources about [a new topic] … not only literature, but people will talk to you about things … I guess, the ability to become conversant, maybe not necessarily an expert, but conversant in areas that you would like to start applying to your research* (Mary)
Project development	Students learn how to ask questions, develop hypotheses, collect and analyze data, and other science practice-type skills as they develop their own projects	*Methods of making sure my research is reproducible. I’ve developed much better habits than I had at the beginning of version control with my code … making sure that I can replicate that.* (Kyle)
Presenting and publishing	Students develop their ability to present and publish their research as they work toward the project goals and the competition deadlines	*For example, with this CASP project, I did one poster presentation … and I also did one oral presentation that … in front of an audience of like a hundred people who were, most of them were probably the professors, so that was a really big opportunity* (Joe)

**Table 5:** Perspectives on the benefits of community challenges

Challenge features	Description	Example
Develop new methods	Participating in challenges allows your research group to be a part of cutting-edge science and the development of new methods	*When I joined the CASP13 conference … and realized how much the field had changed through the introduction of machine learning, I realized that this is the perfect opportunity for us to get involved in this rapidly evolving field* (Dr Della Corte)
Timelines	Community challenges provide real deadlines which can help motivate and move the work along	*It changes things going into the first day, knowing that it starts now … [and] it’s going to last for this long. This is going to be your sole focus for this period … it was able to help focus the questions we were answering.* (Mary)
Clear goals	The community challenge gives the research group clear goals to help focus the work and keep students on track	*I think the goals are very clear … sometimes when you’re trying to work to publish a paper, you don’t actually know what it’s going to end up as … your goals can shift throughout the process … with the competition, you just want to build something that does well and then try it … it’s much simpler goals* (Bill)
Competition and gamification	Knowing your work will be compared with others in a contest can motivate students and it gamifies what normally might be seen as regular work	*There’s a lot of fun in the competition … competing against other groups … it brings a gamification aspect into the more rote process of coming up with a hypothesis, doing experiments, getting results and publishing your paper* (Kyle)
Community integration	Community challenges give students the opportunity to expand their professional networks as they present their findings and interact with other scholars	*[Community projects] make it easy to see how your research compares to others, in a very direct way … you understand the problem a lot better … when you see what other people do, then it’s a lot more meaningful to you* (Joe)
Career advancement	Community challenges give students the opportunity to present their work at a professional conference and can lead to publications in relevant journals	*It really helped me understand what it took to take a paper from results to publication … improving research in the paper until it meets the standards of publication* (Kyle)
