# Supplementary material for: Training undergraduate research assistants with an outcome-oriented and skill-based mentoring strategy
Source: Acta Crystallogr D Struct Biol. 2022 Jul 14;78(Pt 8):936–44. doi: 10.1107/S2059798322005861 (PMC9344475; doi:10.1107/S2059798322005861)
Supplement: Supplementary file 1 [file d-78-00936-sup1.pdf]

STRUCTURAL BIOLOGY

Volume 78 (2022)

Supporting information for article:

Training undergraduate research assistants with an outcome-oriented and skill-based mentoring strategy

Dennis Della Corte, Connor J. Morris, Wendy M. Billings, Jacob Stern, Austin J. Jarrett, Bryce Hedelius and Adam Bennion

S1. Interview Protocol

S1.1. Generic Questions:

1) How did you decide to get involved in research?
    a) What motivated that decision for you?

2) In the beginning, what did you hope to gain, or what outcomes did you expect, from joining a research group and working in that space?

3) What are some of the skills you've developed since starting to do research?

4) Aside from the skills that you feel like you gained, what are some of the other outcomes you’ve seen you because you've participated in research?

5) Tell me how confident you feel doing scientific research or your ability to contribute to the scientific community?

6) When doing research, do you feel like a scientist?
    a) Tell me more about that. For you, what does it mean to be a scientist?

S1.2. Project Specific:

1) How did involvement in the CASP challenge influence your research experience?
    a) What about community projects like that make them a good backdrop to do research?

2) In what ways has involvement in this research group changed your expectations for your career and life after completing your degree? If at all?
